# The Clinical Manifestations of Femoral-Facial Syndrome in an Orthopaedic Patient

**DOI:** 10.1155/2021/6684757

**Published:** 2021-06-14

**Authors:** Abdullah Ghali, Luis Salazar, David Momtaz, Gautham Prabhakar, Preston Richier, Anil Dutta

**Affiliations:** Department of Orthopaedics, UT Health San Antonio, 7703 Floyd Curl Drive, San Antonio, TX, USA

## Abstract

Femoral-facial syndrome (FFS) is an exceedingly rare congenital disorder of unknown etiology related to maternal diabetes during pregnancy. It is characterized by variations of bilateral femoral hypoplasia and facial anomalies. We discuss an interesting case of a 3-year-old girl with FFS with an extensive surgical history who presented to a pediatric orthopaedic clinic with ankle pains and absent femurs. As this disease process is not frequently encountered, it is imperative for the practicing clinician to be aware of the various presentations. In this study, we discuss the different orthopaedic presentations in the literature and discuss various management recommendations.

## 1. Introduction

Femoral-facial syndrome is a rare disorder first described by Daentl et al. in 1975 [1]. Though its formal name has fluctuated since its inception, it was first characterized as “femoral hypoplasia unusual-facies syndrome” with fewer than 70 cases in the current literature to the best of our knowledge [2]. The full syndrome encompasses a wide variety of systems giving this congenital disorder significant clinical heterogeneity. Almost all major systems have been reported in cases of FFS in the literature, though key features include some form of hypoplasia to the bilateral lower extremities and facial anomalies. The cause of FFS has yet to be elucidated [3]. Most cases occur sporadically in females, and a significant association with gestational diabetes has been established [3–6]. Herein, we present a case of femoral-facial syndrome born to a mother with maternal diabetes and preeclampsia. We also discuss FFS etiology, management, and the wide variety of clinical presentations.

## 2. Case Presentation

A 2-day-old female patient, born via c-section at 35 wks due to maternal preeclampsia, was found to have multiple congenital abnormalities including cleft palate, micrognathia, caudal regression, and hypoplastic lower extremities. She was subsequently admitted to the NICU and diagnosed with femoral-facial syndrome related to gestational diabetes. During her 2.5-month admission, she was followed by general, plastic, and orthopaedic surgery receiving a gastrostomy feeding tube, omphalocele repair, and ear molds. Since then, she has also received pressure equalizer tube insertion, jaw reconstruction, and cleft palate repair. Orthopaedics was consulted for her hypoplastic lower extremities for which follow-up 2 weeks after discharge was recommended. She was unfortunately lost to follow-up, and 3 years later, she presented to a pediatric orthopaedic surgery clinic with pain and occasional bleeding on the bony prominences of the ankles while crawling. At follow-up, intellectual disability and psychiatric aggressions characterized by oppositional defiant disorder were documented.

On physical examination, her left elbow lacked full extension and was fused at 90 degrees. She also presented with calluses on the ulnar styloid of her bilateral wrists. The patient had limited pronation and supination bilaterally although she did have full passive range of motion of the shoulder and elbow bilaterally. Her lower extremities presented with no obvious femurs and minimal range of motion at the hip. Her feet were in significant hindfoot varus with prominences along the lateral malleoli. On the right foot, she had preaxial polydactyly of the great toe, while the left foot had both preaxial polydactyly and syndactyly.

**Figure 1:**
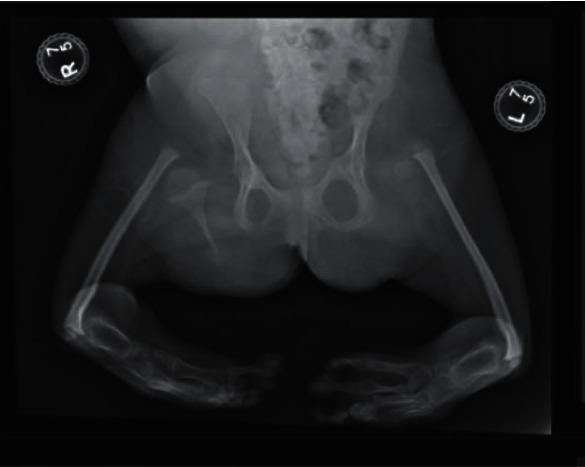
Anteroposterior X-ray showing shortening of bilateral lower extremities with dysplasia of the right femur and bilateral acetabula. The left femur and bilateral fibula are not seen. Partial visualization of the bilateral tibia and the right talus is seen.

**Figure 2:**
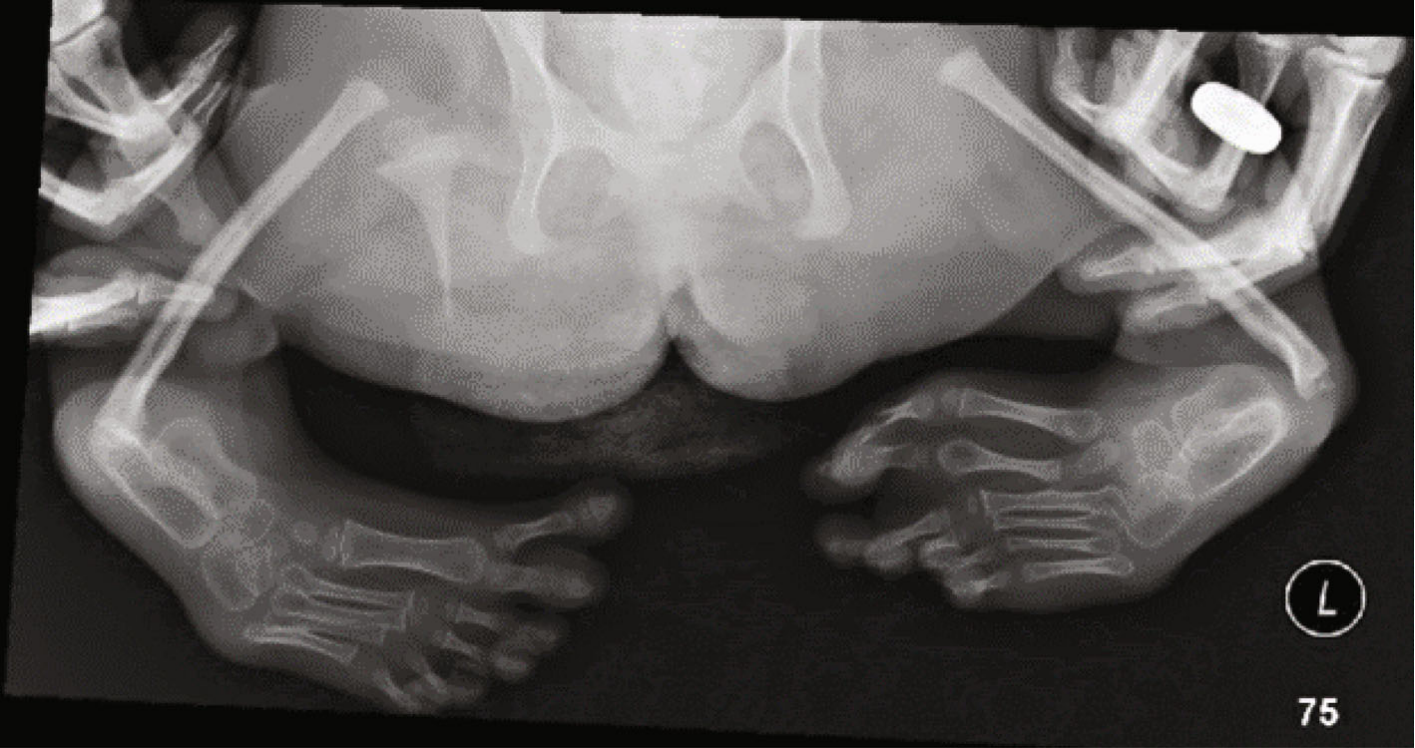
X-ray showing severe deformity of bilateral feet with severe hindfoot varus bilaterally. There are 6 phalanges in the right foot, 2 of them articulating with the first toe metatarsal. There are 6 metatarsals and 6 phalanges in the left foot.

**Figure 3:**
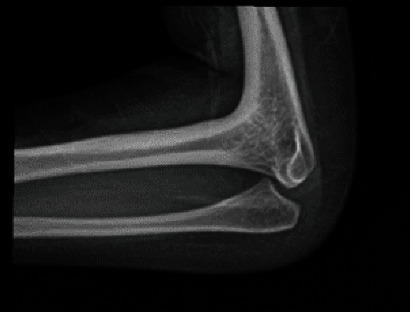
An X-ray of the left elbow demonstrating fusion of proximal radius to the distal humerus.

On plain radiographs, significant foreshortening of the bilateral lower extremities with a hypoplastic right femur and absence of the left femur is demonstrated (Figure 1). Proximal focal femoral deficiency is seen on the right and femur aplasia on the left. She was found to suffer from hindfoot varus bilaterally with 6 phalanges and metatarsals in the right foot (Figure 2). The acetabula are underdeveloped with lumbar and sacral fusion anomalies. The patient also presented with radiohumeral synostosis (Figure 3).

The patient's management was multifaceted. The left radiohumeral synostosis was not removed as it risks the patient from being unable to crawl. She was advised to wait until she was old enough to decide what the best position for hand function is and re-fuse her elbow in further extension and pronation. For the bilateral femoral deficiency and hindfoot varus, rather than lengthening tendons or removing growth centers, the patient was recommended surgery to shorten the tibias and remove the bony prominences that were causing pain. This allowed for future options regarding growth arrest, tendon lengthening, or amputation to be made once we achieved further growth. The patient did not initially pursue surgery and was given a wheelchair.

The patient did not follow up for 3 years and presented two years later with similar pains. At that time, she was referred to the prosthetist for fitting pads with bilateral lower sleeves/orthotics to allow her to bear weight on her hands and feet. Surgery for her bilateral foot polydactyly and syndactyly of 3rd and 4th toes was no longer recommended because any correction would be purely cosmetic.

## 3. Discussion

The unusual presentation of FFS poses a challenge in the timely diagnosis and management for the clinician. It is important to understand this wide variety of presentations to detect FFS early on and tailor management according to each specific presentation. In the current case, rather than lengthening the tendons or removing growth centers, the patient was recommended surgery to shorten the tibias and remove the bony prominences that were causing pain. Ultimately, the decision was made to postpone any surgical intervention until further growth was achieved. Thus, nonoperative care was first initiated, and the patient was given a wheelchair prescription. The current literature regarding FFS is sparse; as such, there is no consensus on management.

Regarding the pathogenesis of this disease process, patients with FFS born to a nondiabetic mother may be linked to a genetic aberration in the terminal end of the 2^nd^ chromosome, 2q37.2 [7]. However, most cases occur sporadically, and several cases have shown autosomal dominant inheritance though multifactorial inheritance seems more likely [3, 8–10]. To the best of our knowledge, autosomal recessive inheritance has been suggested in only one instance [11]. The rare nature of FFS, combined with its incomplete penetrance and variable expressivity has made it difficult to designate the mode of inheritance, with certainty [12]. The disease can also occur spontaneously in families with no known history. In these instances, it seems to be correlated to maternal diabetes mellitus in about a third of patients [4–6]. There also have been links to maternal drug exposure, viral infections, radiation, and oligohydramnios [4, 13]. In their comprehensive literature review, Luisin et al. explore prenatal diagnosis via imaging features that can suggest FFS [14]. They mention that despite advancements in ultrasound, this diagnosis is difficult to make based on defining features and in the absence of genetic or biological markers. Thus, the diagnosis is often made after birth [14].

Clinical manifestations of FFS vary widely and can involve all major systems. In the musculoskeletal system, femoral hypoplasia, ranging from complete agenesis to mild hypoplasia, is a key feature along with facial dysmorphism. Johnson et al. [5] expand the diagnostic criteria so that, for a diagnosis of FFS to be made, femoral hypoplasia along with two out of the four facial features must be present: long philtrum with thin lip, mandible/mouth hypoplasia, upslanting palpebral fissures, or undeveloped nasal alae. When describing the femoral hypoplasia, Darouich et al. describe that it had been reported as symmetric in females, as in our case, but asymmetric in males [2]. Majority of the documented cases report cleft palate, abnormal vertebral size, short limbs, micrognathia and retrognathia, and micromelia (especially concerning the femur) [5, 14]. Clinical manifestations reported as somewhat frequent are talipes equinovarus, xoa vara, abnormal fibular morphogenesis with hypoplasia or aplasia, hip dysplasia of the iliac or ischial bones, enlarged obturator foramen, low set ears with microtia and anotia, thin lips and elongated philtrum, upslanted fissures of the eyes, and fused sacrum and coccyx [5, 14, 15]. The literature also reports very rare findings such as rib fusion, sprengel anomaly, preaxial polydactyly, radioulnar synostosis, or scoliosis [2, 5, 14], with the latter three present in our case.

Other system clinical manifestations can include cardiovascular, neurological, or genitourinary with features such as enlarged penis, ventriculomegaly, polycystic kidney dysplasia, or renal hypoplasia [16]. While genitourinary anomalies are most commonly documented, accounting for 53% of cases, neurological manifestations that impact neural migration may explain the intellectual disability present in 80% of cases [2, 5]. Notably, FFS and caudal regression syndrome are both associated with maternal diabetes and share several similar clinical manifestations [17, 18]. However, facial anomalies help differentiate FFS from caudal dysgenesis. Poon et al. report the case of a Chinese stillborn female with preaxial bilateral polydactyly as well as a bilateral absence of the femurs. Our case seems to be the third of such reports documented in the existing literature [19, 20]. Though 38% of cases are linked to maternal diabetes, the stillborn discussed here was born to a healthy mother who had delivered a normal baby previously [19]. However, an association with high blood glucose levels, at least during gestation, cannot be ruled out as maternal blood glucose levels were measured at 15.5 mmol/l. In another interesting presentation, Vecchio et al. present the case of an infant afflicted with FFS and an autism spectrum disorder (ASD) [21]. Of the 63 reported cases, only 4 have shown some form of central nervous system anomaly. A connection between FFS and ASD could be one other potential feature of FFS [21].

The present study has a wide array of clinical manifestations ranging from common to very rare. Management of the disease has been as wide in its scope as the symptoms itself. That is to say, there is no established standard. García et al. report the case of a 41-week newborn with FFS. The managing physicians opted to take a nonsurgical route to allow for growth [13]. The patient was then scheduled to have surgical bone lengthening of the lower limb at 5 months of age [13]. Similar cases of initial nonsurgical treatment for shortened femurs are reported until the child grows older [14]. Recommended surgical treatments include femoralization of the tibia and use of bilateral hip orthoses [14]. The main purpose of these surgeries was to allow for autonomous movement of the patient [13, 15].

A coordinated holistic effort from a team of specialists is necessary and can prove beneficial in the successful treatment of FFS [14]. Nonsurgical treatments can include a concerted effort from pediatricians, speech pathologists, psychiatrists, physical therapists, and many other specialties depending on the range of symptoms [14, 15]. These interventions can allow for a systematic and comprehensive plan to the child's management and rehabilitation [7, 14]. Furthermore, genetic counseling is recommended to families that have had cases of FFS; as discussed, emerging literature points out a possibility of an autosomal dominant inheritance [7, 8].

## 4. Conclusion

In conclusion, femoral-facial syndrome is a rare congenital disorder with facial, skeletal, and other manifestations. The disease is associated with maternal diabetes, drug use, and viral infections and is also suspected to have an autosomal dominant mode of inheritance. FFS is also known as femoral dysgenesis, bilateral femoral dysgenesis, bilateral-Robin anomaly, and femoral hypoplasia-unusual facies syndrome. The management of the disease has been mostly supportive though some surgical lengthening of the bone, and repositioning of the femur head and acetabulum has been described. The decision to pursue surgical interventions has been largely made with consideration of the patient's age.
